# Effectiveness of non-pharmacological interventions delivered at home for urinary and faecal incontinence with homebound older people: systematic review of randomised controlled trials

**DOI:** 10.1093/ageing/afae126

**Published:** 2024-06-28

**Authors:** Jackie Buck, Julia Fromings Hill, Rachael Collins, Joanne Booth, Jane Fleming

**Affiliations:** School of Health Sciences, Faculty of Medicine and Health Sciences, University of East Anglia, Norwich, UK; St Bartholomew’s Hospital, Barts Health NHS Trust, West Smithfield, London EC1A 7BE, UK; School of Health Sciences, Faculty of Medicine and Health Sciences, University of East Anglia, Norwich, UK; School of Health Sciences, Faculty of Medicine and Health Sciences, University of East Anglia, Norwich, UK; Research Centre for Health, School of Health and Life Sciences, Glasgow Caledonian University, Glasgow, UK; Cambridge Public Health Interdisciplinary Research Centre, University of Cambridge, Cambridge, UK

**Keywords:** urinary incontinence, faecal incontinence, homebound, older people, systematic review

## Abstract

**Introduction:**

Incontinence is a common, distressing condition, most prevalent in older people. There is an unmet need for effective interventions to support continence. This review focuses on non-pharmacological interventions to reduce incontinence among homebound older people. Aim: to identify interventions with potential to be delivered by care workers, nurses or family members in a person’s home.

**Methods:**

Multiple databases were searched until 15 September 2023 for randomised controlled trials reporting home-based interventions for incontinence for older people (≥65 years) living at home. Two reviewers independently screened titles, abstracts and papers against inclusion criteria, then assessed for the Risk of Bias (RoB2). A third reviewer resolved the discrepancies. Primary data were extracted and synthesised.

**Results:**

A full-text review of 81 papers identified seven eligible papers (1996–2022, all USA), including *n* = 636 participants (561 women and 75 men). Two studies focusing on multicomponent behavioural interventions showed benefit, as did one study of transcutaneous tibial nerve stimulation self-administered through electrode-embedded socks. Three, which included cognitively impaired people, reported improvement with toileting assistance programmes, but the effects were not all significant. Results were inconclusive from a study examining the effects of fluid intake adjustments. Interventions were delivered by nurses, three in collaboration with family caregivers. No faecal incontinence interventions met the criteria.

**Conclusion:**

There is scant evidence for continence supporting interventions delivered in older people’s own homes. With an ageing population often reliant on family or social care workers well-placed to support continence promotion and policy drives for services to support older people remaining at home, this evidence gap needs addressing.

## Key Points

- It may be possible to reduce urinary incontinence in older homebound adults using behavioural interventions.
- Only one trial using a non-invasive technology delivered at home was identified. Other approaches with potential for delivery at home, but so far only tested in younger age-ranges or settings, warrant further research.
- There have been no randomised controlled trials of interventions to support homebound older adults with faecal incontinence.
- Older homebound adults, particularly men, are rarely included in research into new interventions for incontinence.

## Introduction

Problems with continence, which manifest as different subtypes of urinary and/or faecal incontinence, are distressing yet common conditions that increase in prevalence with ageing and impact on both individuals and those living with them [1–4]. Incontinence is often poorly assessed, diagnosed and managed, with older adults least likely to be offered evidence-based treatments [5, 6]. Incontinence is more prevalent among people with multiple co-morbidities, including obesity [7], depression [8], mobility impairment [9] and dementia [10], and is associated with loneliness and social isolation [11], poor quality of life [12], caregiver stresses [13] and moving into care [14].

Frequently under-reported due to embarrassment, seeing continence problems as normal ageing and being unaware of treatment possibilities, many people struggle to cope at home without seeking help [15, 16]. Prevalence estimates vary across different populations and settings, with urinary incontinence (UI) consistently more commonly reported than faecal, affecting not only the frailest but also fitter community-living older people [17, 18]. As the older age groups, and particularly the ‘oldest old’, are now the fastest-growing section of the population, and not only in the developed world [19], the prevalence of incontinence is rising [5] and understanding the needs of the older people affected is a global issue [20]. Pharmacological solutions can play a helpful role in managing some types of incontinence, for instance, prostate problems, nocturia or over-active bladder. However, given the risks of polypharmacy in older people [21, 22], particularly for the oldest and frailest [23, 24] who are most likely to be housebound [25–27], and the uncertainty about the long-term side-effects of anti-cholinergic medications [28, 29], alternative approaches to supporting continence in this population are necessary.

Evidence suggests that incontinence is remediable for many people using simple lifestyle and behavioural approaches [30, 31]. To date, the bulk of research on incontinence in older people has focused on people in hospital or in care homes settings, where prevalence is more easily estimated than in community settings, and intervention effects are more readily quantified in a more clearly defined target group. Yet only 2.3% of people aged 65 years and older live in care homes (even among those aged ≥85 years, this proportion is only 10.2% [32]), and acute hospital stays are usually so short [33] that any meaningful intervention requires continuation into community settings after discharge home.

Group interventions delivered in clinic settings for people living with incontinence have been reported; however, these often have relatively high attrition rates [30]. Reasons include the severity of incontinence, inconvenient location or time and dislike of groups [34]. Stigma associated with services for incontinence may also be a factor [35].

Homebound older adults are significantly disadvantaged in terms of access to healthcare services, and there has been a paucity of research on this increasing group of vulnerable adults [36]. Given the association of incontinence with physical, mental and social consequences, there is a need to explore home-delivered interventions, particularly for people who are unable or unwilling to attend group sessions. The rationale for the review was informed by discussions, led by reviewer JF, with the local Public Involvement in Research into Ageing and Dementia group.

The aim of this systematic review is to determine the effectiveness of home-delivered interventions for urinary and/or faecal incontinence in community-living older adults.

## Methods

We conducted a systematic search of the literature to identify randomised controlled trials of non-pharmacological interventions delivered in the home to improve incontinence in older people living at home in non-institutionalised settings.

### Search strategy and selection criteria

The search strategy was developed in collaboration with an academic librarian and was founded on two initial scoping searches, which helped to inform and refine the search terms. The search terms were generated from the following concepts. Population: older people age 65; condition: UI, faecal incontinence; interventions: conservative, non-pharmacological, non-surgical; context: delivered in the home. Search strategies combine free text terms, limited to title and abstract-only searches, with keyword/subject heading searches. The full search strategy for Medline (via Ovid) is shown in Supplementary Information Appendix 1 and was adapted for searches in CINAHL, PsycInfo (via EbscoHOST), Embase and EMCare (via OVID). Grey literature was sought from COPAC, EThOS, OpenGrey and Proquest using a modified search strategy. Hand searches, including reference lists of relevant review papers and all included papers, completed the review.

Articles were included in the review if they described the results of randomised controlled trials of home-based conservative interventions to support urinary or faecal continence in people aged 65 and older living in non-institutional community settings. We excluded papers that trialled any pharmacological interventions. Interventions delivered in care homes, out-patient or primary care settings or community clinics were excluded, even if the follow-up was conducted in the home.

Results of searches up to 15 September 2023 were imported into EndNote and duplicates removed. Titles, abstracts and selected full text papers were screened independently by two members of the review team (JBu, RC, JF and JFH). At each point, discrepancies in screening decisions were discussed within the team, and an inclusive approach was adopted to allow for the maximum possibility of capturing relevant articles. The methodological quality of each included paper was separately evaluated by two of the three reviewers (JBu, RC and JF) using the Cochrane Risk of Bias 2 tool [37]. In order to present a comprehensive overview of the literature in this area and to minimise bias, we included all studies regardless of quality.

Two review team members (JBu and JF) independently extracted data from the included studies (data collection templates are available from authors on request). A narrative synthesis of the findings was conducted to determine the common and distinguishing features of the studies, and pooling of outcomes in a meta-analysis was undertaken where data allowed.

A full review protocol was registered with the PROSPERO International Prospective Register of Systematic Reviews 2019 (CRD42019141664), https://www.crd.york.ac.uk/prospero/display_record.php?RecordID=141664.

This review is reported in accordance with the Preferred Reporting Items for Systematic Review and Meta-analysis (PRISMA) checklist for quality (Supplementary Information Appendix 2).

## Results

**Figure 1:**
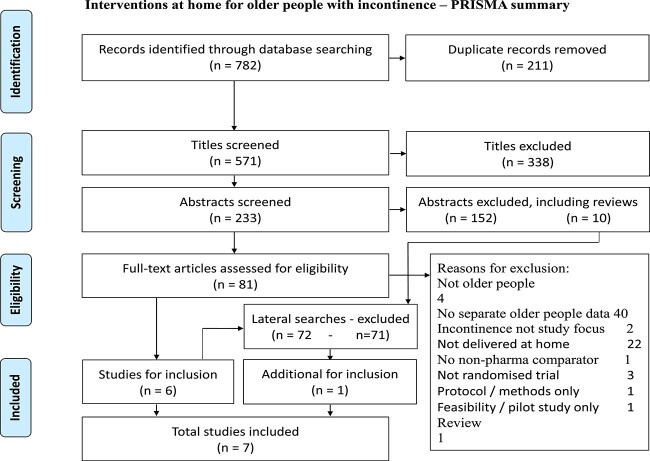
PRISMA flow diagram.

The PRISMA flow diagram (Figure 1) summarises the process that led to the identification of seven papers from six studies that met inclusion criteria. The characteristics of the included papers are found in Table 1. All studies were conducted in the United States, two of which included only women. All studies targeted UI; three specified the type (overactive bladder, stress/urgency/mixed and functional incontinence). The number of participants ranged from 19 to 218, and their mean age ranged from 68 to 83 years old.

**Table 1:** Description of studies included in the review.

Authors and year	Population and type of incontinence	Sample size and sex	Mean age (range)	Risk of bias	Intervention	Control	Duration	Assessment(s)	Main outcome(s)
*Multicomponent behavioural interventions*									
McDowell *et al*., 1999 [28]	Homebound women and men aged ≥60 Any urinary incontinence	105 W = 95 M = 10	77 (61–97)	Low	Behavioural therapy: 8x weekly nurse practitioner visits covering • Pelvic floor muscle exercise • Urge and stress strategies • Bladder retraining	Cross-over trial: waiting list control group started intervention after 8 wks of social visits every 1–2 wks by nurse practitioner	8 wks	9–10 wks: (*n* = 48 Ix, *n* = 40 cross-over Cx → Ix, *n* = 45 Cx) 3, 6, 9 and 12 mo (n not reported)	Frequency of incontinent episodes
Dougherty *et al*., 2002 [29]	Women aged ≥55 living at home in rural areas Any urinary incontinence	218 W = 218 M = 0	68 (55–95)	Some concerns	Behaviour management techniques: nurse visits, individualised and progressing through. . . • Self-monitoring: fluids and caffeine intake, timing of intake and voiding, diet promoting bowel regularity (2–4 wks) • Bladder retraining (6–8 wks) • Pelvic floor muscle exercise with biofeedback (12 wks)	Feedback given on information obtained at the baseline visit—not treatment nor promoting treatment—same number of follow-ups every 6 mo	6–24 mo	6 mo (*n* = 78 Ix, *n* = 69 Cx) 12 mo (*n* = 59 Ix, *n* = 52 Cx) 18 mo (*n* = 34 Ix, *n* = 31 Cx) 24 mo (23 Ix, *n* = 23 Cx) Any of above (*n* = 178)	Frequency of incontinent episodes Quantity of urine loss (g) Quality of life
*Toileting assistance programmes (care-giver assisted)*									
Jirovec and Templin, 2001 [30]	Memory-impaired older people living at home Functional urinary incontinence	118 W = 82 M = 36	80 (range not reported)	Some concerns	• Individualised scheduled toileting and fluid management: Nurse home visit every 2 mo teaching caregivers strategies + phone-call every month discussing progress	Nurse phone-call every month just ‘friendly visits’ with no continence focus	6 mo	6 mo (*n* = 44 Ix, *n* = 30 Cx)	Frequency of incontinent episodes
Engberg *et al*., 2002 [31]	Cognitively impaired homebound women and men aged ≥60 Urinary incontinence	19 W = 13 M = 6	83 (69–93)	Some concerns	• Individualised prompted voiding: 8 nurse practitioner visits once a wk	Cross-over trial: waiting list control group started intervention after 8 wks of social visits every 1–2 wks by nurse practitioner	8 wks	9–10 wks: (*n* = 6 Ix, *n* = 9 cross-over Cx → Ix, *n* = 10 Cx)	Frequency of incontinent episodes
Colling *et al*., 2003 [32]	Cognitively impaired women and men aged ≥55 i) caregiver-dependent ii) living alone Urinary incontinence	78 W = 63 M = 15 i) n = 59 ii) n = 19	76 (range not reported)	High	• Pattern Urge-Response Toileting: researcher home visit teaching toileting regime + weekly follow-up phone-calls	Cross-over trial: waiting list control group started intervention after 15 wks of only data collection	6 wks	3 wks—after baseline run-in, 9 wks—after 6 wk intervention (*n* = 31 Ix, *n* = 24 cross-over Cx → Ix, *n* = 24 Cx) 12 and 15 wks (*n* not reported)	Frequency of incontinent episodes Quantity of urine loss
*Transcutaneous Tibial Nerve Stimulation (TTNS)*									
Cava and Orlin, 2022 [33]	Women and men with ICIQ-LUTS (F/M) score > 60 living at home Over-active bladder +/− urge urinary incontinence	40 W = 32 M = 8	68 (52–85)	Low	TTNS using electrodes embedded in a conventional sock + battery-operated attachable stimulation device—30 min sessions once weekly—self-administered at home	Identical electrode embedded sock + identical looking battery-operated sham device which lit up when on but no stimulation—30 min sessions once weekly—self-administered at home	12 wks	12 wks (*n* = 20 Ix, *n* = 18 Cx)	*1^0^ outcome:* ‘success’ = ≥50% ↓ urgency voids +/− UI or ≥ 30% ↓ 24-hour frequency *Secondary* *2^0^ outcome:* quality of life
*Fluid intake adjustment*									
Dowd *et al.*, 1996 [34]	Women aged >50 with UI living at home	58 W = 58 M = 0	70 (52–89)	High	Adjustment of fluid intake—2 Ix groups: increase and decrease intake	Maintain fluid intake	5 wks	Weekly x5 wks 3 mo (*n* = 10 decrease, *n* = 8 maintain, *n* = 14 increase)	Frequency of incontinent episodes

W = women, M = Men, Ix = Intervention, Cx = Control, ICIQ-LUTS (F/M) = International Consultation on Incontinence Questionnaire Lower Urinary Tract Symptoms Female or Male module, 1^0^ = primary, 2^0^ = secondary.

Most interventions could be classified into two groups: two studies included multicomponent behavioural interventions (MBIs) [38, 39] and three were toileting assistance programmes (TAPs) [40–42]. Both MBI studies [38, 39] targeted urgency and mixed UI through bladder training and targeted stress incontinence through pelvic floor muscle exercises (PFMEs), and both incorporated lifestyle changes as well [39]. The TAPs reported were prompted voiding [41], habit training [42] and individualised scheduled toileting [40]. The results reported in 1999 [38] and 2002 [41] by McDowell, Engberg and colleagues were from different groups within the same trial. Two interventions were only investigated in single studies, one of transcutaneous tibial nerve stimulation [43] and one exploring the effects of adjusting fluid intake [44].

Recruitment approaches varied. Three studies recruited members of the public [40, 42, 44], one used both referral to a medical centre and advertising to recruit [43], [38, 41] one recruited participants who were in receipt of home care identified by nursing staff (results reported in two papers [38, 41]) and one did not describe recruitment [39]. Not all papers comment on recruitment challenges, but Colling and colleagues [42] reported great difficulty recruiting adequate numbers for the trial and altering the sampling strategy significantly to recruit sufficient participants to achieve power. McDowell, Engberg and colleagues [38, 41] also reported high participation refusal rates with consent by only 24% of those eligible. Reasons for this included the duration of the study (15 months) and the need to complete a daily diary.

Reported attrition rates of the studies ranged from 16% to 45%. The most frequently cited reason for withdrawal was the deteriorating health of either the participant or caregiver. In the study by Jirovec and colleagues, 19 of 44 participants who did not complete the study moved into residential care [40]. Attrition of fifteen of 25 older women [39] and 14 of 44 caregivers [40] occurred due to intervention demands considered to be too high in terms of time and effort.

The studies were of variable quality, with two at low risk of bias [38, 43], three indicating some cause for concern about bias [39–41] and two at high risk of bias [42, 44]. Two studies gave power calculations, one of which [43] recruited to target, but the other [42] only met the sample size by revising inclusion criteria, accepting participants living alone with no full-time caregiver despite the intervention targeting caregivers. In all other studies, there were recruitment difficulties and resultantly small sample sizes. Stratified randomisation achieved well-matched intervention and control groups in the two papers reporting different interventions with sub-samples of the same study [38, 41], groups were comparable despite no stratification in two studies [39, 43], in one study [42], there were marked differences between intervention and control groups and two studies provided no details for comparison of randomised group characteristics [40, 44].

All interventions were delivered by nurses, and the three studies, which centred around TAPs, also included caregivers in the delivery of the intervention. People who were cognitively impaired were excluded from four of the six studies [38, 39, 43, 44]. Bladder diaries were used to collect data in all studies, but the duration varied considerably. Some required diaries to be kept throughout the intervention period (5 weeks [44] and 8 weeks [38]) to allow for adjustment of the intervention during the trial. In one trial, data collection was repeated 6 months apart [39]. Others collected data pre and postintervention for periods ranging from 3 days [42, 43] to 1 [40] or 2 [41] weeks.

A variety of outcome measures were used, preventing meta-analysis of effect sizes. Although the two MBI trials and one of the TAP trials included one outcome measure in common (daily UI episode frequency), mean differences in changes of frequency at follow-up between intervention and control group participants were reported with no measures of uncertainty, precluding an estimation of the pooled effect from both studies. Other objective outcome measures included percentage change in daytime UI frequency [41], 24-hour urinary frequency [39, 43], volume of incontinent loss of urine [39, 42], percentage of wet day-time pad-checks [41], frequency of urgency voids [43] and proportion of voiding episodes incontinent [40]. Participant-reported outcomes included two different quality of life scales [39, 43], a subjective report of the severity of urine loss [39] and caregiver burden [41].

All studies reported benefits for participants who received interventions, either as a reduction in the frequency of incontinence episodes or a decrease in the volume of involuntary urine loss (Table 2).

**Table 2:** Findings from included studies.

Authors and year	Key findings	Intervention group outcomes	Control group outcomes	Significance of difference
*Multicomponent behavioural interventions*				
McDowell *et al.*, 1999 [28]	• Higher % of Ix group than Cx group completely continent by 8wks • Greater ↓ in urinary incontinent episodes/day in Ix than Cx group • Men and those using walking aids were less likely to benefit • Those who did ≥30 exercises/day, had lower depression scores or lived with someone were more likely to benefit	15% *Initial Ix group:* ↓55% *All Ix including Cx→Ix group:* ↓58%	Not reported ↓ 15%	Not reported *P* = .006
Dougherty *et al*., 2002 [29]	*At all follow-ups Ix group compared with Cx group (showing @24 m f/up):* • Greater ↓ in incontinent episodes/24 h, greatest at 24 m • Greater ↓ in urine loss/24 h, greatest at 24 m • Greater ↑ in subjective severity of urine loss • Greater ↑ reported quality of life, greatest at 24 m	↓70% ↓61% ↑47 (higher score = better control) ↓30% (lower score = better QoL)	↓16% ↑184% ↑19 ↓13%	*P* = .0001 *P* = .0006 *P* < .05 *P* = .0025
*Toileting assistance programmes*				
Jirovec and Templin, 2001 [30]	• Higher % with ↓ in incontinent episodes	64% (28/44)	50% (15/30)	*P* = <.05
Engberg *et al.*, 2002 [31]	• Greater ↓ in day-time incontinent episodes (difference not significant, by intention to treat or per protocol analyses) • Greater ↓ in % of daytime wetness on pad-checking (difference not significant, by intention to treat or per protocol analyses)	*ITT: ↓*50% *PP: ↓*60% *ITT:* ↓50% *PP:* ↓50%	↓37% ↓35%	*P* = .27 *P* = .1 *P* = .35 *P* = .24
Colling *et al.*, 2003 [32]	• Greater ↓ in incontinent episodes/24 hrs (non-significant) • Greater ↓ in incontinent urine loss volume/24 hrs	*Initial Ix group:* ↓19% *Cx→Ix group:* ↓9% *Initial Ix group:* ↓39% *Cx→Ix group:* ↓56%	↓ 12% ↓ 3.5%	*P* = .23 *P* = .21 *P* < .02 *P* < .05
*Transcutaneous Tibial Nerve Stimulation*				
Cava and Orlin, 2022 [33]	• Higher % achieved treatment success • Greater improvement in self-reported quality of life	80% −29.1 (SD 16.5)	39% −17.7 (SD 12.8)	*P* = .02 *P* < .001
*Fluid intake adjustment*				
Dowd *et al.*, 1996 [34]	• No significant associations between UI and either fluid/caffeine intake • Fluid intake protocol adherence low • Reported most valuable learning from study participation: need to increase fluid intake	1–5 wks*:* inconclusive 3 mo: not reported by study arm	n/a n/a	Not reported Not reported

MBIs effectively reduced episodes of UI by more than two per day in both the MBI trials [38, 39], statistically significant reductions in both. In the only TTNS study [43], more than twice as many who were treated with TTNS than with sham stimulation met treatment success criteria, with more than 50% higher improvements in this trial’s secondary outcome, quality of life. Two other studies, both TAP trials, reported effect sizes that reached statistical significance in at least one outcome [40, 42]. For the study with the smallest sample (*n* − 19) [41] and those with the shortest duration (5 weeks [44] and 6 weeks [42]), effects were not [41] or not all [42] statistically significant, or significance was not reported [44]. Participants in the intervention groups of the studies, which included subjective outcome measures, all reported more improvements in these than control group participants: better quality of life [39, 43] and greater perceived bladder control [39]. Most caregivers supporting their care recipients with a prompted voiding programme reported perceived improvements in the form of fewer and smaller incontinent episodes and said they would continue to follow the programme after the study ended [41].

## Discussion

The review aimed to identify interventions for incontinence in older people with potential for effective delivery in the home by care workers, nurses or family members. The limited evidence found for this care context showed varying degrees of effectiveness for three types of interventions: (i) MBIs, combining lifestyle changes, self-monitoring, PFME and bladder training; (ii) a wearable device intervention, transcutaneous tibial nerve stimulation delivered through a sock and (iii) TAPs, such as prompted voiding or habit training, where the aim was not to change bladder function but to avoid or minimise episodes of UI by supporting carers to manage the older person’s voiding. Only the first two of these included studies with a low risk of bias and achieved significantly improved incontinence and quality of life outcomes. Study results indicate MBIs are effective in reducing episodes of UI and the severity of leakage among community-dwelling older women [38, 39], but there is insufficient evidence to determine individual component contributions. Concerns about bias were higher for TAP studies, from which evidence of effectiveness was less consistent and weaker, and likewise for the only included study of fluid intake adjustment [44].

There is an extensive body of evidence for all three types of incontinence interventions summarised above and for some of their components, showing their effectiveness in settings other than at home, e.g. particularly for PFME in nurse or physiotherapist-led groups [45–47] or TTNS in clinics [48, 49]. The high-quality evidence of efficacy for PFME in young and middle aged women [50] and in men undergoing prostatectomy [51, 52] is beginning to extend to older [50, 53] and even frail older populations [54], but rarely from interventions conducted at home. Similarly, TAPs have largely been investigated in the context of long-term care, where, in theory, formal caregivers are continuously available to deliver the intervention [55, 56]. Although the only fluid adjustment study in our review [44] reported no significant findings, a recent systematic review of caffeine and fluid interventions unrestricted by intervention setting or age criteria found these could be effective for over-active bladder symptoms [57].

This review’s focus on randomised trials of interventions delivered at home adds to learning from previous reviews of interventions for incontinence among older people, which included non-randomised studies or programmes in institutional settings [17, 30, 31, 54, 56, 58, 59]. Its strengths include its inclusion of multiple databases using comprehensive search strategies and extensive lateral searches. No language limits were applied, but no relevant studies in any language other than English met the inclusion criteria.

A number of review limitations are noteworthy: the included studies were all from the USA, and all but one [43] were published 15–23 years ago, so their applicability to current healthcare provision and practice across other countries’ healthcare systems may be unconvincing. Study quality varied; the high risk of bias that some papers scored, largely due to confusing reporting or a lack of detail in study procedure descriptions, led to discomfort when deciding on inclusion. The type of UI was not identified in any study, and outcomes focused on quantifiable episodes of UI or volume of leakage, with patient reported outcome measures used in only two studies, which included quality of life [39, 43] and perceived leakage changes [39] as secondary outcomes.

It was conspicuous that the studies included only a small number of men. Although UI affects women more than men, overlooking the profound impact of incontinence on men by limiting trials to only include women denies opportunities for improvement of a condition that men also find isolating and disabling [60–62].

A notable review finding was the lack of randomised controlled trials to test interventions delivered in the home for people with faecal incontinence. Given the sensitive nature of the condition and its associated social stigma and co-morbidities, this would seem like a prime area for further research and practice development. Excluded studies in slightly younger populations suggest two approaches with potential for investigation with older people at home—biofeedback [63] and tibial nerve stimulation [64].

Other continence promotion interventions have only been tested in younger age ranges, including some studies with younger old people excluded from this review by its ≥65 age limit. This limit had been set with the age-range of house-bound older people in mind, who are often even older. Evidence for effectiveness in older age could not be extracted for a number of promising interventions with potential for delivery at home that appear to merit further research, specifically with older people. These included weighted vaginal cones [65], transvaginal electrical stimulation [65, 66], a weight loss programme [67], a comparison of a specialist nurse service with self-help leaflets [68], a computer-based continence promotion programme [69], internet versus leaflets for non-face-to-face pelvic floor exercise instruction [70] and a mobile app [71].

The marked worsening of incontinence among control group participants in one study [39] highlights the need for effective interventions. Although recruitment was challenging and attrition was high in some of the studies, those that achieved significant benefits illustrate the scope for further use of successful approaches. Encouragingly, secondary analysis [72] comparing homebound and non-homebound participants in one of the included MBI studies [38] found benefits were no less among the homebound. Given that homebound older adults are more likely to be in poorer health with increased functional disability than the general population [25–27], these results are encouraging and warrant further research. The challenges of study recruitment and intervention adherence, particularly carer fatigue, even for well-supported adults, as seen in the studies in this review, suggest that incontinence treatment options are challenging. Future studies to address the evidence gaps this review has highlighted will need to be developed with the involvement of housebound older people affected by continence problems and of those supporting them. Co-design approaches will be vital to ensuring that further research can minimise these challenges through testing acceptable and feasibly sustainable interventions.

There is an increasing awareness of the environmental impact of urinary and faecal incontinence products, which are usually single-use items or, if reusable, may require frequent laundering at relatively high temperatures [73]. Striving to deliver holistically sustainable continence care [74] further increases the need for interventions, which could lead to a reduction in the use of these products.

Ensuring that older people have access to evidence-based interventions that can be delivered at home will depend on the experience, attitudes and appropriate training of community nurses and care staff [75–79].

## Conclusion

Robust evidence supporting conservative in-home interventions for older people with UI was found in a limited number of small studies. Randomised controlled trials of interventions for management of faecal incontinence have not been conducted in this population and setting. These and other approaches, so far only tested in younger age ranges or in institutional or other group settings, but with potential for delivery at home, warrant further research to build the evidence base for continence promotion among older people. Research that tailors urinary and faecal incontinence interventions to the specific needs of homebound women and men is imperative to improve the quality of life in this growing population.

## Supplementary Material

aa-23-1995-File003_afae126

## References

[1] Perry S , Shaw C, Assassa P et al. An epidemiological study to establish the prevalence of urinary symptoms and felt need in the community: the Leicestershire MRC incontinence study. J Public Health 2000;22:427–34. 10.1093/pubmed/22.3.427 11077920

[2] Perry S , Shaw C, McGrother C et al. Prevalence of faecal incontinence in adults aged 40 years or more living in the community. Gut 2002;50:480–4. 11889066 10.1136/gut.50.4.480 PMC1773171

[3] Menees SB , Almario CV, Spiegel BMR et al. Prevalence of and factors associated with fecal incontinence: results from a population-based survey. Gastroenterology 2018;154:1672–1681.e3. 29408460 10.1053/j.gastro.2018.01.062 PMC6370291

[4] Santini S , Andersson G, Lamura G. Impact of incontinence on the quality of life of caregivers of older persons with incontinence: a qualitative study in four European countries. Arch Gerontol Geriatr 2016;63:92–101. 26620553 10.1016/j.archger.2015.10.013

[5] Harari D , Husk J, Lowe D et al. National audit of continence care: adherence to National Institute for Health and Clinical Excellence (NICE) guidance in older versus younger adults with faecal incontinence. Age Ageing 2014;43:785–93. 24850541 10.1093/ageing/afu056

[6] Mamza JB , Geary R, El-Hamamsy D et al. Variation in surgical treatment advice for women with stress urinary incontinence: a study using clinical case vignettes. Int Urogynecol J 2020;31:1153–61. 32253488 10.1007/s00192-020-04295-4 PMC7270981

[7] Reigota RB , Pedro AO, de Souza Santos Machado V et al. Prevalence of urinary incontinence and its association with multimorbidity in women aged 50 years or older: a population-based study. Neurourol Urodyn 2016;35:62–8. 25358890 10.1002/nau.22679

[8] de Vries HF , Northington GM, Bogner HR. Urinary incontinence (UI) and new psychological distress among community dwelling older adults. Arch Gerontol Geriatr 2012;55:49–54. 21601929 10.1016/j.archger.2011.04.012 PMC4084656

[9] Monz B , Pons ME, Hampel C et al. Patient-reported impact of urinary incontinence--results from treatment seeking women in 14 European countries. Maturitas 2005;52:S24–34. 16297579 10.1016/j.maturitas.2005.09.005

[10] Miu DK , Lau S, Szeto SS. Etiology and predictors of urinary incontinence and its effect on quality of life. Geriatr Gerontol Int 2010;10:177–82. 20446932 10.1111/j.1447-0594.2009.00574.x

[11] Stickley A , Santini ZI, Koyanagi A. Urinary incontinence, mental health and loneliness among community-dwelling older adults in Ireland. BMC Urol 2017;17:29. 28388898 10.1186/s12894-017-0214-6 PMC5385037

[12] Bartoli S , Aguzzi G, Tarricone R. Impact on quality of life of urinary incontinence and overactive bladder: a systematic literature review. Urology 2010;75:491–500. 19962738 10.1016/j.urology.2009.07.1325

[13] Talley KMC , Davis NJ, Peden-McAlpine C et al. Navigating through incontinence: a qualitative systematic review and meta-aggregation of the experiences of family caregivers. Int J Nurs Stud 2021;123:104062. 34461378 10.1016/j.ijnurstu.2021.104062

[14] Scheibl F , Farquhar M, Buck J et al. When frail older people relocate in very old age, who makes the decision? Innov Aging 2019;3:igz030. 10.1093/geroni/igz030 PMC7127322 32274424

[15] Horrocks S , Somerset M, Stoddart H et al. What prevents older people from seeking treatment for urinary incontinence? A qualitative exploration of barriers to the use of community continence services. Fam Pract 2004;21:689–96. 15528285 10.1093/fampra/cmh622

[16] Alimohammadian M , Ahmadi B, Janani L et al. Suffering in silence: a community-based study of fecal incontinence in women. Int J Colorectal Dis 2014;29:401–6. 24322737 10.1007/s00384-013-1809-3

[17] Teunissen TA , van den Bosch WJ, van den Hoogen HJ et al. Prevalence of urinary, fecal and double incontinence in the elderly living at home. Int Urogynecol J Pelvic Floor Dysfunct 2004;15:10–3. 14752592 10.1007/s00192-003-1106-8

[18] Milsom I , Gyhagen M. The prevalence of urinary incontinence. Climacteric 2019;22:217–22. 30572737 10.1080/13697137.2018.1543263

[19] World Health Organisation . Ageing and Health. World Health Organisation. https://www.who.int/news-room/fact-sheets/detail/ageing-and-health(2022, date last accessed).

[20] Agnew R , Booth J. Promoting urinary continence with older people: a selective literature review. Int J Older People Nurs 2009;4:58–62. 20925803 10.1111/j.1748-3743.2008.00158.x

[21] Davies LE , Spiers G, Kingston A et al. Adverse outcomes of polypharmacy in older people: systematic review of reviews. J Am Med Dir Assoc 2020;21:181–7. 31926797 10.1016/j.jamda.2019.10.022

[22] Hilmer SN , Gnjidic D. The effects of polypharmacy in older adults. Clin Pharmacol Ther 2009;85:86–8. 19037203 10.1038/clpt.2008.224

[23] Wang R , Chen L, Fan L et al. Incidence and effects of polypharmacy on clinical outcome among patients aged 80+: a five-year follow-up study. PLoS One 2015;10:e0142123. 26554710 10.1371/journal.pone.0142123 PMC4640711

[24] Cesari M . How polypharmacy affects frailty. Expert Review of Clinical Pharmacology 2020;13:1179–81. 32985932 10.1080/17512433.2020.1829467

[25] Celeiro ID , Santos-del-Riego S, García JM. Homebound status among middle-aged and older adults with disabilities in ADLs and its associations with clinical, functional, and environmental factors. Disabil Health J 2017;10:145–51. 27461941 10.1016/j.dhjo.2016.06.006

[26] Negrón-Blanco L , de Pedro-Cuesta J, Almazán J et al. Prevalence of and factors associated with homebound status among adults in urban and rural Spanish populations. BMC Public Health 2016;16:1–11. 10.1186/s12889-016-3270-z PMC4946192 27422021

[27] Herr M , Latouche A, Ankri J. Homebound status increases death risk within two years in the elderly: results from a national longitudinal survey. Arch Gerontol Geriatr 2013;56:258–64. 23116977 10.1016/j.archger.2012.10.006

[28] Dmochowski RR , Thai S, Iglay K et al. Increased risk of incident dementia following use of anticholinergic agents: a systematic literature review and meta-analysis. Neurourol Urodyn 2021;40:28–37. 33098213 10.1002/nau.24536 PMC7821204

[29] Mehdizadeh D , Hale M, Todd O et al. Associations between anticholinergic medication exposure and adverse health outcomes in older people with frailty: a systematic review and meta-analysis. Drugs Real World Outcomes 2021;8:431–58. 34164795 10.1007/s40801-021-00256-5 PMC8605959

[30] Kilpatrick KA , Paton P, Subbarayan S et al. Non-pharmacological, non-surgical interventions for urinary incontinence in older persons: a systematic review of systematic reviews. The SENATOR project ONTOP series. Maturitas 2020;133:42–8. 32005422 10.1016/j.maturitas.2019.12.010

[31] Stenzelius K , Molander U, Odeberg J et al. The effect of conservative treatment of urinary incontinence among older and frail older people: a systematic review. Age Ageing 2015;44:736–44. 26112402 10.1093/ageing/afv070

[32] Office for National Statistics UK . Life expectancy in care homes in England and Wales 2021 - 2022. Office for National Statistics, 2023.

[33] Ewbank L, Thompson J, McKenna H et al. NHS Hospital Bed Numbers: Past, Present and Future. London: The King's Fund, 2021.

[34] Kincade JE , Johnson TMI, Ashford-Works C et al. A pilot study to determine reasons for patient withdrawal from a pelvic muscle rehabilitation program for urinary incontinence. J Appl Gerontol 1999;18:379–96.

[35] MacInnes CL . Why women leave therapy for stress incontinence. Nurs Times 2008;104:50–3. 18979961

[36] Qiu WQ , Dean M, Liu T et al. Physical and mental health of homebound older adults: an overlooked population. J Am Geriatr Soc 2010;58:2423–8. 21070195 10.1111/j.1532-5415.2010.03161.x PMC3044592

[37] Sterne JAC , Savović J, Page MJ et al. RoB 2: a revised tool for assessing risk of bias in randomised trials. BMJ 2019;366:l4898. 31462531 10.1136/bmj.l4898

[38] McDowell BJ , Engberg S, Sereika S et al. Effectiveness of behavioral therapy to treat incontinence in homebound older adults. J Am Geriatr Soc 1999;47:309–18. 10078893 10.1111/j.1532-5415.1999.tb02994.x

[39] Dougherty MC , Dwyer JW, Pendergast JF et al. A randomized trial of behavioral management for continence with older rural women. Res Nurs Health 2002;25:3–13. 11807915 10.1002/nur.10016

[40] Jirovec MM , Templin T. Predicting success using individualized scheduled toileting for memory-impaired elders at home. Res Nurs Health 2001;24:1–8. 11260580 10.1002/1098-240x(200102)24:1<1::aid-nur1001>3.0.co;2-r

[41] Engberg S , Sereika SM, McDowell BJ et al. Effectiveness of prompted voiding in treating urinary incontinence in cognitively impaired homebound older adults. J Wound Ostomy Cont Nurs 2002;29:252–65. 10.1067/mjw.2002.127207 12510471

[42] Colling J , Owen TR, McCreedy M et al. The effects of a continence program on frail community-dwelling elderly persons. Urol Nurs 2003;23:117–31. 12778826

[43] Cava R , Orlin Y. Home-based transcutaneous tibial nerve stimulation for overactive bladder syndrome: a randomized, controlled study. Int Urol Nephrol 2022;54:1825–35. 35622269 10.1007/s11255-022-03235-z PMC9137441

[44] Dowd TT , Campbell JM, Jones JA. Fluid intake and urinary incontinence in older community-dwelling women. J Community Health Nurs 1996;13:179–86. 8916607 10.1207/s15327655jchn1303_5

[45] Subak LL , Quesenberry CP, Posner SF et al. The effect of behavioral therapy on urinary incontinence: a randomized controlled trial. Obstet Gynecol 2002;100:72–8. 12100806 10.1016/s0029-7844(02)01993-2

[46] Kim H , Suzuki T, Yoshida Y et al. Effectiveness of multidimensional exercises for the treatment of stress urinary incontinence in elderly community-dwelling Japanese women: a randomized, controlled, crossover trial. J Am Geriatr Soc 2007;55:1932–9. 17944890 10.1111/j.1532-5415.2007.01447.x

[47] Sherburn M , Bird M, Carey M et al. Incontinence improves in older women after intensive pelvic floor muscle training: an assessor-blinded randomized controlled trial. Neurourol Urodyn 2011;30:317–24. 21284022 10.1002/nau.20968

[48] Booth J , Connelly L, Dickson S et al. The effectiveness of transcutaneous tibial nerve stimulation (TTNS) for adults with overactive bladder syndrome: a systematic review. Neurourol Urodyn 2018;37:528–41. 28731583 10.1002/nau.23351

[49] Araujo TG , Schmidt AP, Sanches PRS et al. Transcutaneous tibial nerve home stimulation for overactive bladder in women with Parkinson's disease: a randomized clinical trial. Neurourol Urodyn 2021;40:538–48. 33326648 10.1002/nau.24595

[50] Choi H , Palmer MH, Park J. Meta-analysis of pelvic floor muscle training: randomized controlled trials in incontinent women. Nurs Res 2007;56:226–34. 17625461 10.1097/01.NNR.0000280610.93373.e1

[51] Johnson EE , Mamoulakis C, Stoniute A et al. Conservative interventions for managing urinary incontinence after prostate surgery. Cochrane Database Syst Rev 2023;2023. 10.1002/14651858.CD014799.pub2. PMC10112049 37070660

[52] Tienforti D , Sacco E, Marangi F et al. Efficacy of an assisted low-intensity programme of perioperative pelvic floor muscle training in improving the recovery of continence after radical prostatectomy: a randomized controlled trial. BJU Int 2012;110:1004–10. 22332815 10.1111/j.1464-410X.2012.10948.x

[53] Talley KMC , Wyman JF, Bronas U et al. Defeating urinary incontinence with exercise training: results of a pilot study in frail older women. J Am Geriatr Soc 2017;65:1321–7. 28248418 10.1111/jgs.14798 PMC5478439

[54] Talley KMC , Wyman JF, Shamliyan TA. State of the science: conservative interventions for urinary incontinence in frail community-dwelling older adults. Nurs Outlook 2011;59:215–220.e1. 21757078 10.1016/j.outlook.2011.05.010 PMC3846433

[55] Roe B , Milne J, Ostaszkiewicz J et al. Systematic reviews of bladder training and voiding programmes in adults: a synopsis of findings on theory and methods using metastudy techniques. J Adv Nurs 2007;57:3–14. 17184370 10.1111/j.1365-2648.2006.04098.x

[56] Roe B , Ostaszkiewicz J, Milne J et al. Systematic reviews of bladder training and voiding programmes in adults: a synopsis of findings from data analysis and outcomes using metastudy techniques. J Adv Nurs 2007;57:15–31. 17184371 10.1111/j.1365-2648.2006.04097.x

[57] Park J , Lee H, Kim Y et al. Effectiveness of fluid and caffeine modifications on symptoms in adults with overactive bladder: a systematic review. Int Neurourol J 2023;27:23–35. 37015722 10.5213/inj.2346014.007 PMC10073005

[58] Shamliyan T , Wyman J, Bliss DZ et al. Prevention of urinary and fecal incontinence in adults. Evid Rep Technol Assess 2007;161:1–379. PMC4781595 18457475

[59] Göransson C , Larsson I, Carlsson IM. Art of connectedness: value-creating care for older persons provided with toileting assistance and containment strategies—a critical interpretive synthesis. J Clin Nurs 2023;32:1806–20. 35034383 10.1111/jocn.16216

[60] Buczak-Stec E , König H-H, Hajek A. How does the onset of incontinence affect satisfaction with life among older women and men? Findings from a nationally representative longitudinal study (German ageing survey). Health Qual Life Outcomes 2020;18:1–8. 10.1186/s12955-020-1274-y PMC6985999 31992311

[61] Cheng MC , Liu SP, Chuang YC et al. Prevalence and impacts of male urinary incontinence on quality of life, mental health, work limitation, and health care seeking in China, Taiwan, and South Korea (LUTS Asia): results from a cross-sectional, population-based study. Investig Clin Urol 2022;63:71–82. 10.4111/icu.20210259 PMC8756147 34983125

[62] Esparza AO , Tomás MÁC, Pina-Roche F. Experiences of women and men living with urinary incontinence: a phenomenological study. Appl Nurs Res 2018;40:68–75. 29579501 10.1016/j.apnr.2017.12.007

[63] Thomas GP , Dudding TC, Bradshaw E et al. A pilot study to compare daily with twice weekly transcutaneous posterior tibial nerve stimulation for faecal incontinence. Colorectal Dis 2013;15:1504–9. 24118972 10.1111/codi.12428

[64] Xiang X , Sharma A, Patcharatrakul T et al. Randomized controlled trial of home biofeedback therapy versus office biofeedback therapy for fecal incontinence. Neurogastroenterol Motil 2021;33:e14168. 34051120 10.1111/nmo.14168

[65] Seo JT , Yoon H, Kim YH. A randomized prospective study comparing new vaginal cone and FES-biofeedback. Yonsei Med J 2004;45:879–84. 15515199 10.3349/ymj.2004.45.5.879

[66] Barroso JC , Ramos JG, Martins-Costa S et al. Transvaginal electrical stimulation in the treatment of urinary incontinence. BJU Int 2004;93:319–23. 14764129 10.1111/j.1464-410x.2004.04608.x

[67] Skelly J . A behavioural weight-loss programme reduced urinary incontinence more than an education programme in overweight and obese women. Evid Based Nurs 2009;12:110. 19779077 10.1136/ebn.12.4.110

[68] Wagg AR , Barron D, Kirby M et al. A randomised partially controlled trial to assess the impact of self-help vs. structured help from a continence nurse specialist in women with undiagnosed urinary problems in primary care. Int J Clin Pract 2007;61:1863–73. 17764454 10.1111/j.1742-1241.2007.01552.x

[69] Boyington AR , Dougherty MC, Phetrasuwan S. Effectiveness of a computer-based system to deliver a continence health promotion intervention. J Wound Ostomy Cont Nurs 2005;32:246–54. 10.1097/00152192-200507000-00009 16030464

[70] Sjostrom M , Umefjord G, Stenlund H et al. Internet-based treatment of stress urinary incontinence: 1- and 2-year results of a randomized controlled trial with a focus on pelvic floor muscle training. BJU Int 2015;116:955–64. 25683075 10.1111/bju.13091 PMC4690161

[71] Asklund I , Nystrom E, Sjostrom M et al. Mobile app for treatment of stress urinary incontinence: a randomized controlled trial. Neurourol Urodyn 2017;36:1369–76. 27611958 10.1002/nau.23116

[72] Engberg S , Sereika SM. Effectiveness of pelvic floor muscle training for urinary incontinence comparison within and between nonhomebound and homebound older adults. J Wound Ostomy Continence Nurs 2016;43:291–300. 27163683 10.1097/WON.0000000000000227

[73] Macaulay M , Murphy C, Fader M et al. Sustainability 2: are sustainable continence products a realistic option? Nurs Times 2020;116:32–29.

[74] Vaittinen T , Koljonen K, Tella S et al. Holistically sustainable continence care: a working definition, the case of single-used absorbent hygiene products (AHPs) and the need for ecosystems thinking. Proc Inst Mech Eng H 2023;9544119231188860. 37655850 10.1177/09544119231188860 PMC11318206

[75] Månsson-lindström A , Dehlin O, Isacsson A. Urinary incontinence in primary health care 1. Perceived knowledge and training among various categories of nursing personnel and care units. Scand J Prim Health Care 1994;12:169–74. 7997694 10.3109/02813439409003694

[76] Månsson-lindström A , Dehlin O, Isacsson A. Urinary incontinence in primary health care 2. Care routines and consequences — perception of various categories of nursing personnel and care units. Scand J Prim Health Care 1994;12:175–9. 7997695 10.3109/02813439409003695

[77] Bignell V , Getliffe K. Clinical guidelines for the promotion of continence in primary care: community nurses' knowledge, practice and perceptions of their role. Prim Health Care Res Dev 2006;2:173–86.

[78] Yuan HB , Williams BA, Liu M. Attitudes toward urinary incontinence among community nurses and community-dwelling older people. J Wound Ostomy Cont Nurs 2011;38:184–9. 10.1097/WON.0b013e31820af394 21326113

[79] McCann M , Kelly A-M, Eustace-Cook J et al. Community nurses’ attitudes, knowledge and educational needs in relation to urinary continence, continence assessment and management: a systematic review. J Clin Nurs 2022;31:1041–60. 34296482 10.1111/jocn.15969

